# Severe acneiform eruption associated with TNFRSF13B mutation: Clinical response to adalimumab

**DOI:** 10.1016/j.jdcr.2025.06.056

**Published:** 2025-08-19

**Authors:** Ka Wei Katty Joo Hu, Claudio Karsulovic, Lia Hojman

**Affiliations:** a Facultad de Medicina Clínica Alemana de Santiago, Universidad del Desarrollo, Santiago, Chile; b Investigation in Dermatology and Autoimmunity-IDea Lab, Instituto de Ciencias e Innovación en Medicina, Universidad del Desarrollo, Santiago, Chile; c Rheumatology Section, Internal Medicine Department, Facultad de Medicina Clínica Alemana de Santiago, Universidad del Desarrollo, Santiago, Chile; d Dermatology Section, Surgery Department, Facultad de Medicina Clínica Alemana de Santiago, Universidad del Desarrollo, Santiago, Chile

**Keywords:** acneiform eruption, adalimumab, autoinflammatory syndrome, case report

## Introduction

Acneiform eruptions can present in the context of acne vulgaris, making diagnosis challenging when atypical features or systemic symptoms are present.^1^ Although acne vulgaris is characterized by comedones and is highly prevalent among adolescents, some syndromes with immune dysregulation can mimic or exacerbate acneiform lesions. These autoinflammatory syndromes—such as Pyoderma gangrenosum, Acne, and Hidradenitis suppurativa (PASH), Pyogenic Arthritis, Pyoderma gangrenosum and Acne (PAPA), and Synovitis, Acne, Pustulosis, Hyperostosis, and Osteitis (SAPHO)—are associated with mutations in genes regulating innate immunity and often manifest with sterile inflammation, nodules, pustules, and involvement of intertriginous or atypical sites. A high index of suspicion is required when systemic symptoms or treatment resistance are present. Mutations in TNFRSF13B, which encodes a protein called TACI are typically linked to common variable immunodeficiency (CVID) and not classically associated with autoinflammatory syndromes.^2^^,^^3^ We report an uncommon case of a teenager with a heterozygous TNFRSF13B mutation and a severe acneiform eruption with systemic inflammatory features that responded dramatically to tumor necrosis factor-alpha (TNF-α) blockade—raising the possibility of a previously unrecognized inflammatory phenotype associated with this variant.

## Case report

**Fig 1:**
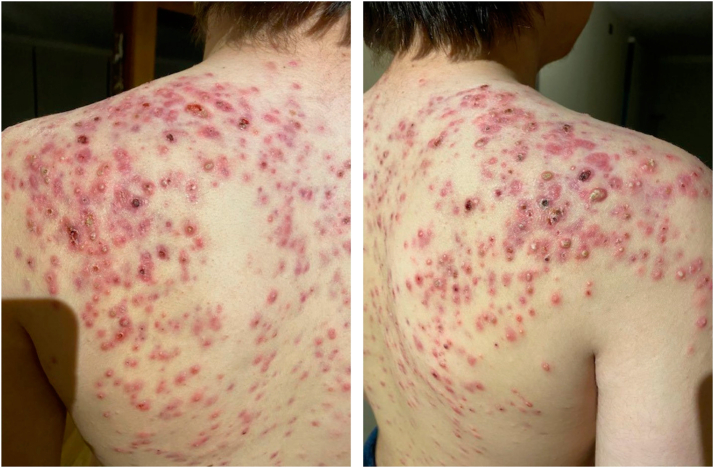
Severe acneiform lesions involving trunk and intertriginous areas.

A 13-year-old male with a history of central precocious puberty treated with triptorelin presented with a 2-month history of nodulocystic acne on the trunk. He developed systemic symptoms including fatigue, febrile sensations, and anorexia. On physical examination, it was revealed that he had severe acneiform lesions consistent with acne conglobata on the trunk (Fig 1) and additional furuncle-like lesions on the groin, buttocks, and scalp.

Initial treatment with oral prednisone 30 mg/day led to significant improvement, but tapering and transitioning to isotretinoin resulted in a flare characterized by worsening inflammation and increased systemic symptoms. Given the involvement of intertriginous areas, systemic features, and unresponsiveness to conventional acne treatment, an autoinflammatory syndrome was suspected. Laboratory investigations revealed markedly elevated inflammatory markers, including a C-reactive protein level of 7.72 mg/dL (reference, 0.10-0.50 mg/dL), interleukin (IL) 6 of 31.4 pg/mL (reference, 0-3.4 pg/mL), soluble IL-2 receptor (sCD25) at 932 U/mL, and ferritin at 373 ng/mL (reference, 15-201 ng/mL).

Treatment was changed to methylprednisolone 12 mg/day, sulfasalazine 500 mg twice a day, trimethoprim-sulfamethoxazole (3 times weekly), and topical clobetasol 0.05%. After 1 month, he reported a 50% reduction in pain and a 60% reduction in inflammation. Active lesions decreased to 10%, mostly leaving scars on the trunk and mild residual activity in intertriginous areas.

A genetic panel (Invitae, GeneMetrics) targeting autoinflammatory syndromes did not identify known pathogenic variants but revealed a heterozygous variant in TNFRSF13B, associated with CVID. No history of recurrent infections or immune deficiency was noted.

**Fig 2:**
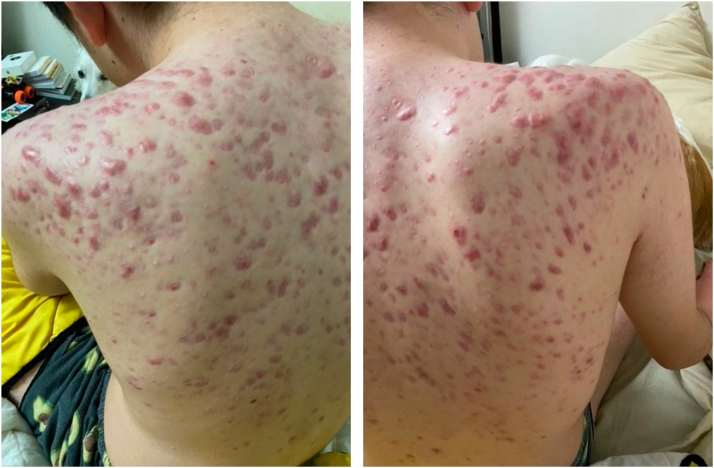
Complete resolution of acneiform lesions after 3 doses of adalimumab.

Given persistent inflammation, adalimumab 40 mg every 2 weeks was initiated while maintaining low-dose methylprednisolone. One week after the first injection, only residual inflamed lesions and crusts remained. After 3 doses, the patient had no active lesions (Fig 2), and methylprednisolone was tapered further.

## Discussion

Autoinflammatory acneiform eruptions may be difficult to distinguish from severe acne vulgaris, especially when classical features such as comedones are present.^2^ Syndromes like Pyogenic Arthritis, Pyoderma gangrenosum and Acne (PAPA), Pyoderma gangrenosum, Acne, and Hidradenitis suppurativa (PASH), Synovitis, Acne, Pustulosis, Hyperostosis, and Osteitis (SAPHO), and Tumor Necrosis Factor Receptor–Associated Periodic Syndrome (TRAPS) involve mutations in genes regulating innate immunity and may present with recurrent fever, pustules, joint symptoms, and sterile inflammation.^4^, ^5^, ^6^, ^7^

Mutations in TACI, typically associated with CVID, are not known to cause autoinflammatory syndromes. However, TACI modulates B cell survival and regulates T–independent immune responses. Its dysfunction has been associated not only with hypogammaglobulinemia but also with immune hyperactivation in certain contexts.^8^ The presence of this variant in a patient with severe inflammation and dramatic response to anti–TNF therapy raises the possibility of an inflammatory role for this mutation.

This patient may represent an incomplete form of PASH syndrome, which includes pyoderma gangrenosum, acne, and hidradenitis suppurativa. Although the patient did not develop pyoderma gangrenosum, early intervention may have prevented full phenotypic expression. Treatment regimens for PASH typically include corticosteroids, immunosuppressants, antibiotics, and biologics targeting IL-1 or TNF-α. Our patient responded rapidly to TNF-α blockade, supporting a diagnosis within this spectrum.^6^ Interestingly, the patient continues to have facial acne vulgaris despite complete resolution of the truncal and intertriginous lesions, highlighting the likely autoinflammatory nature of the latter.

A similar case reported by Feola et al^9^ described a PASH syndrome with an atypical Mediterranean fever gene (MEFV) mutation that responded well to adalimumab, further supporting the therapeutic rationale.

Although the clinical pattern and therapeutic response strongly suggest an autoinflammatory component, certain limitations must be acknowledged. A skin biopsy was not performed; however, given the characteristic pattern of lesions, systemic inflammatory features, and the rapid clinical response to immunomodulatory therapy, histopathological confirmation was not deemed essential for diagnosis or management. Additionally, although the TNFRSF13B variant raises suspicion of a contributory role in inflammation, a direct pathogenic link cannot be confirmed in the absence of functional studies or larger case series. As such, this report should be viewed as hypothesis-generating, highlighting a potentially novel phenotype that warrants further exploration.

Future studies are needed to evaluate if TNFRSF13B variants may contribute to a broader spectrum of sterile inflammatory diseases, particularly in genetically predisposed individuals with atypical acneiform presentations.

## Conclusion

This case highlights the importance of considering autoinflammatory processes in patients with severe acneiform eruptions, particularly when systemic symptoms and treatment resistance are present. It also suggests the existence of a novel inflammatory phenotype possibly linked to TNFRSF13B mutations. The potential link between TNFRSF13B mutations and inflammation warrants further investigation.

## Conflicts of Interest

None disclosed.
